# Public Telemedicine Policy in Brazilian Unified Health System: An Impact Analysis

**DOI:** 10.3390/ijerph21060657

**Published:** 2024-05-22

**Authors:** Angela Maria de Oliveira, Marcos Aurélio Pereira Valadão, Benjamin Miranda Tabak

**Affiliations:** Escola de Políticas Públicas e Governo, Fundação Getulio Vargas (FGV/EPPG), Brasília 70830-020, Brazil; marcos.valadao@fgv.br

**Keywords:** public policies, telemedicine, teleconsultation, eHealth, TeleSUS

## Abstract

There are several difficulties in evaluating interventions seeking to promote public health policies. In this article, we analyzed the promotion of the use of telemedicine during COVID-19 in Brazil. Using the random promotion method with instrumental variables, we showed that the policy of promoting telemedicine was adequate, with intense use of this type of care. Our results showed that telemedicine works if it is encouraged in the population. We contributed to the discussion of public health policies and their impact on the population’s health in times of health crisis, such as during the COVID-19 pandemic.

## 1. Introduction

Advances in information and communications technologies (ICTs) have provided the emergence of tools with immense potential to address some challenges in delivering health services in an accessible, affordable, and high-quality manner for both developed and developing countries. According to the Global Observatory for eHealth, telemedicine uses ICTs to overcome geographic barriers and increase access to health services, which is particularly beneficial for rural and underserved communities in developing countries [1].

In 2019, the WHO launched its Global Strategy on Digital Health. The document, published in 2021, unifies the concepts that had been pulverized to designate the areas of action, covering other uses of digital technologies for health, such as the Internet of Things, artificial intelligence, big data, and robotics [2].

As for eHealth, the WHO considers the safe and cost-effective use of ICTs in health support and related fields, including health services and surveillance, literature, education, knowledge, and health research [2]. In this context, the Ministry of Health understands that the unification of all the concepts of ICT applications in health, including eHealth, telemedicine, telehealth and mobile health, reduces fragmentation, and encompasses emerging technologies in ICT under the umbrella of digital health [3].

Qualitative data surveys carried out by entities such as Paulista Medicine Association (APM) [4] collected information throughout the national territory, not presenting specific data from regions. However, according to the entity, the adoption of telemedicine/teleconsultation tools was well received by the medical class, hospital organizations, and public hospitals linked to both federal and private universities. Among the population, surveys revealed that more than half of respondents know what telemedicine is, and half of them have already received teleconsultation and support using the tool, especially among health plan users.

This article assesses the impact of the telemedicine policy in Brazil’s Unified Health System (SUS) during the COVID-19 pandemic between April 2020 and April 2021. To do so, we adopted the concept of the Global Observatory for eHealth [1]. We considered the TeleSUS and teleconsultation applications made available by DataSus, the SUS IT department linked to the Secretariat Office for Strategic and Participatory Management of the Ministry of Health.

The article structure involves searching for evidence in the DataSus health database and renowned entities such as IBGE and CETIC.br. The first step was to collect all relevant information on the topic and establish indicators and expected results through research questions. The second was the screening and selection of publications pertinent to the development of the work. The third was preparation: tabulating the data and comparing the results of the quantitative analysis with the qualitative research already carried out, given the policy’s objectives.

Curioso et al. [5] discuss the difficulties of implementing telemedicine in Peru and its importance during the COVID-19 pandemic. Hung et al. [6] studied the characteristics of telemedicine users during the COVID-19 pandemic in the United States. They concluded that the use of telemedicine was higher among the elderly, blacks, college-educated, and disabled. Wu et al. [7] studied telemedicine in Taiwan and found it is less popular among older and less educated people due to perceived social and psychological risks. Hosseinzadeh et al. [8] show how telemedicine in Bangladesh was perceived as satisfactory during the COVID-19 pandemic.

We contributed to this literature by using a novel approach to show that the incentives provided by the Brazilian government to use telemedicine during the COVID-19 pandemic were effective, with many people adhering to telemedicine treatments. We also contributed to the discussion on evaluating public policies when there are limited data. We showed that even with limited data, we can evaluate whether public policies have had a positive effect on telemedicine’s use, controlling for errors by using the random promotion as an instrumental variable approach, as proposed by Gertler et al. [9].

To the best of our knowledge, this article is a pioneering evaluation of a public policy to encourage the use of telemedicine, with a need for more data. Using a random promotion approach as an instrumental variable, we showed that the policy of promoting health through telemedicine was effective. This result contributes to the design of policies to stimulate telemedicine, which is essential in countries as large as Brazil, where there is a significant shortage of medical professionals in smaller towns and less populated regions.

## 2. Theoretical Reference

### 2.1. Telemedicine Policy in the SUS (Single Health System)

Before the Federal Constitution of 1988 (CF-88), medical assistance in Brazil was a public service under the responsibility of the National Institute of Medical Assistance of Social Security—INAMPS, created by Law No. 6439/1977. According to information from the Ministry of Health, assistance was provided only to workers linked to social security [10].

The Constitution of the Federative Republic of Brazil [11], enacted on 5 October 1988, in its article 196, established the universalization of the Brazilian health system. However, the first steps towards its regulation came with Decree No. 99,060/1990 [12] for transferring the former INAMPS from the Ministry of Social Security to the Ministry of Health. Nevertheless, it was only with the signing of Law No. 8080/1990 [13] that the Unified Health System (SUS) was instituted. The next important step was in 1993, with Law No. 8689, extinguishing INAMPS and transferring its competencies to the federal, state, and municipal instances that manage the SUS, enshrining the universal right to health and the unification/decentralization of responsibility for the management of health services [14] to states and municipalities.

According to the Ministry of Health [15], the SUS is one of the world’s largest and most complex public health systems. The three principles that guide the SUS are universalization, equity, and completeness.

SUS national coverage comprises the population estimated by the IBGE [16] for 2021 at 213.4 million. At the extremes, Roraima has 554.6 thousand inhabitants, and São Paulo has more than 46 million. Of the municipalities, only 49 have more than 500,000 inhabitants; 23 are capital cities. The 27 capitals have 50 million inhabitants, equivalent to 23.86% of the country’s total population in 2020 [16]. The SUS governance structure encompasses representatives from the union, states, and municipalities with the distribution of shared responsibilities [17], as shown in Table 1 below.

The CNS published the Charter of the Rights of Health Users, which brings together six basic principles of citizenship. It highlights that “every citizen has the right to orderly and organized access to health systems” [17].

### 2.2. Telemedicine Policy

The WHO edited a resolution in 2005 that recommended strategies for strengthening health systems through eHealth to member states. Following WHO guidance, Brazil created the National Telehealth Program through Ordinance No. 35/2007, revoked in 2010 and redefined by Ministry of Health Ordinance No. 2546/2011, which expanded the program and is now called the National Program Telehealth Brazil Networks. The services available are teleconsulting, telediagnosis, tele-education, and formative second opinion. The telehealth centers carry out the policy focusing on supporting permanent education to qualify primary care teams and increase resolution [18].

Teleconsultation, however, was not authorized in Brazil. The Federal Council of Medicine (CFM) published Resolution No. 1643/2002 [19], allowing telemedicine only in emergencies.

In 2018, the CFM released Resolution No. 2227 [20], revising the 2002 Resolution and allowing teleconsultation. In 2019, it published a new Resolution No. 2228/2019 [21], revoking No. 2227/2018 and restoring the effectiveness of No. 1643/2002. Also, in 2019, the federal government published Decree No. 9795, dictating the competencies of the Ministry of Health, including the areas of telehealth, telemedicine, and digital health [22]. It is incumbent upon the Ministry’s Executive Secretariat Office to “formulate, coordinate and monitor actions and strategies related to the National Policy on Digital Health and Telehealth of the SUS, within the Ministry of Health”. To this end, the Department of Digital Health was created, linked to the Secretariat Office, whose functions include “formulating, planning, coordinating, supervising, monitoring and evaluating the implementation of the National Policy on Digital Health and Telehealth in the SUS” [22].

The Ministry of Health’s actions demonstrated an evolution in expanding ICT tools to optimize primary care services in the SUS. However, with the emergence of the pandemic caused by the new coronavirus (SARS-CoV-2), COVID-19, and the need to maintain distance, in addition to other health measures, the federal government sanctioned Law No. 13,989/2020 as a matter of urgency, while the pandemic lasts, allowing the use of telemedicine for both public and private care [23]. The CFM continued to have the competence to regulate the practice of telemedicine. In general, countries have sought telemedicine to improve health services [24], and telemedicine has proven to be efficient in combating the COVID-19 pandemic [25,26,27].

### 2.3. Policy Instruments and Tools

The Ministry of Health (MS) has the National Health Plan (PNS) and the Pluriannual Plan (PPA) for planning public health policies within the federal public administration. Policy needs and resource availability must be contained in state, municipal, and federal district health plans. The CNS elaborates on the guidelines and priorities for the construction of the plans. The PNS 2020–2023 [28], aligned with the PPA 2020–2023 [29], defined strategic objectives to expand the use of technologies in the SUS. The purpose was to “improve the management of the SUS to guarantee access to equitable and quality health goods and services” to produce 3,100,000 diagnostic reports through telehealth, increasing the computerization of primary healthcare teams to 92%, and connecting 27 federation units to the National Health Data Network—RNDS [30], which was created to be the national platform for the interoperability of health data in Brazil. Therefore, the topic was already on the MS agenda and had been evolving since 2007.

MS Ordinance No. 467/2020 regulated and operationalized the use of telemedicine (teleconsultation, electronic certificate, and prescription signed with an ICP-Brazil digital certificate) while the measures to deal with the pandemic lasted [31].

MS/DataSus made the TeleSUS tool available for pre-clinical healthcare to clarify the infected population and when to seek face-to-face care. According to information on the Ministry of Health website [32], the objective was to help with the population’s home isolation and prevent the depletion of face-to-face health services. The entry channels to the proposed health services are the ChatBot Service, the Audible Response Unit Service (URA), the Preclinical Care Service (SAPC), and remote monitoring. The APP Coronavirus SUS was made available for pre-clinical care, Dial Health 136, Online Chat at https://coronavirus.saude.gov.br/ (accessed on 29 March 2021), and an exclusive WhatsApp channel at the number (61) 9938-0031. In parallel, MS and DataSus were already developing the RNDS implementation project, which represents the basis of the Connect SUS Program, which, in turn, is part of the Digital Health Strategy [33].

Another vital measure in addition to teleconsultation was the issuance of medical certificates and prescriptions electronically, made possible by a tool developed by the National Institute of Information Technology (ITI) in conjunction with the CFM and the Federal Council of Pharmacy (CFF). The Digital Document Validator website allows patients, pharmacists, and physicians to validate digital prescriptions, certificates, medical reports, and exam requests [34].

Hospitals linked to federal universities also played an essential role in strengthening call center services during the COVID-19 pandemic by developing and making tools available according to each unit’s strategies [35].

## 3. Materials and Methods

The policy addressed in this paper was implemented as urgent and, therefore, did not follow the recommended planning steps: ex-ante analysis with a clear definition of objectives, indicators and targets, and adequate monitoring for a subsequent evaluation of results. Due to this, to fulfill the objective outlined in this study (to elaborate on the impact assessment of the telemedicine policy in the Brazilian Unified Health System between April 2020 and April 2021), some critical indicators considered essential needed to be defined to guide data collection, as described in Table 2. It should be noted that the authors defined the indicators based on the research context and information availability.

### 3.1. Technique Used and Parameterization

Gertler et al. [9] described the technique used in this work, which is based on random promotion as an instrumental variable.

The parametrization includes the following: the target public benefiting from the policy is SUS users. Implementation took place simultaneously across the country from April 2020 onwards. The analysis will be retrospective when the program’s impact assessment occurs after its implementation, generating treatment and comparison groups a posteriori. The instrumental variable method helps to evaluate programs with universal coverage, partial compliance, or voluntary enrollments, so an instrumental variable can be described as something outside the individual’s control, influencing their probability of participating in the program, but not being associated with the participant’s characteristics [9].

Considering, in this case, that the policy presents universal eligibility, and that the administrator has no control over who participates or not in the initiative, the chosen approach was “random promotion”, which acted as an instrumental variable [9]. As part of the approach, the encouragement for the random group to participate in the program originated from the strategies to stimulate the policy of social isolation and combat the spread of COVID-19. It was not related to the characteristics of the groups of participants. The focus of this evaluation was to estimate the impact of telemedicine on the SUS and not the impact of the promotion and adherence strategy on the final results. It is important to note that this study did not seek to analyze the criteria used to motivate the general population to encourage the use of resources made available by SUS units for primary healthcare. The dissemination of actions to combat the coronavirus pandemic was the responsibility of the union, states, and municipalities, each in its scope of action. The websites of each agency and social media were the resources most used by all to disseminate information and use the tools available, in addition to telephone contact. According to Mélo et al. [36], 77.8% of Brazilian states announced teleconsultation services. Of these, the vast majority (77.8%) offered the service via a telephone call, 55.6% via videoconference, and 70.4% adopted other tools (WhatsApp, Gmail, Google Meet, Skype, Zoom, Online Chat, Facetime, Hangout, and home apps). The effectiveness of the actions was verified by analyzing the quantitative data of the selected indicators.

The authors used the following questions as a guide in conducting the analysis. The answers will appear in the Conclusion section of this study depending on the research results:

1. What is the representative impact of telemedicine on the care of the SUS population between April 2020 and April 2021?
2. What is the effect of implementing telemedicine/teleconsultation in reducing hospital procedures? In what proportion?
3. What is the impact of telemedicine on the population of underprivileged regions/areas?
4. What are the main stakeholders’ expectations?
5. What is the perception of policy participants regarding the user experience?
6. Should telemedicine be maintained after the pandemic period?

### 3.2. Selection of Groups

The groups selected for analysis were composed of the states of the north (Acre, Amapá, Amazonas, Pará, Roraima, Rondônia, and Tocantins) and northeast (Alagoas, Bahia, Ceará, Maranhão, Paraíba, Pernambuco, Piauí, Rio Grande do Norte, and Sergipe) regions. These states were chosen because most of the population is concentrated in extreme poverty (monthly household income of up to R$145.00 per capita) and poverty (monthly income between R$145 and R$420 per capita), with a high SUS dependency. According to the IBGE [37], 64.7% of users of primary healthcare services have a per capita household income of less than one minimum wage, and 32.4% earn from one to three minimum wages.

In 2018, the northeast region contained 47.9% of Brazil’s poverty, followed by the north region with 26.1% [38]. The IBGE/Continuous PNAD survey 2019 [39] shows that the north region had 8.6% of the country’s resident population and the northeast region 27.2%.

According to the adopted methodology, there are three types of participants: those who participate if encouraged (“complier” or “enroll-if-assigned”), those who “never participate”, and those who “always participate”. Compliers are those potentially interested in joining the program. However, for various reasons, they did not, and, in this case, they may need to obtain more information or the correct incentive to do so [9].

After the quantitative collection of the number of telemedicine/teleconsultations authorized by the SUS and registered in the DataSus database, we found that some states did not participate in the program (never participates or participates if motivated but did not receive an incentive; as such, they can be assumed as the group not assigned for treatment), others participated little or started later (participates if encouraged), and some were immediately engaged (always participates). The states of Maranhão, Piauí, Amazonas, Acre, and Roraima had no record of procedures performed by teleconsultation until that moment. Therefore, for this research, we deduce that they were not participating in the program and did not develop actions (or they were ineffective) to motivate the population to use the available resources.

Adopting the random promotion approach, based on Gertler et al. [9], two groups of eight elements with similar characteristics were created: group 1—motivated to participate: Alagoas, Bahia, Ceará, Paraíba, Pernambuco, Piauí, Rio Grande do Norte, and Sergipe; group 2—little/not motivated to participate: Acre, Amapá, Amazonas, Maranhão, Pará, Roraima, Rondônia, and Tocantins. In this case, however, as the analysis was based on ex-post data, there was no intervention in the participants’ motivation, which, supposedly, was stimulated according to the strategy adopted by each state/municipality, which, according to Mélo et al. [36], was uneven for several reasons, such as the availability of infrastructure, trained personnel, and the number of specialized professionals, among others. The state of Maranhão joined group 2 to balance the count.

The total participation percentage in group 1 was 87.5% (seven states: AL–BA–CE–PB–PE–RN–SE), and in group 2 it was 50% (four states: RO–PA–AP–TO). The Δ% was 37.5% (87.5–50%). To promote the impact evaluation, the first step was to find the result of each group according to the chosen indicators (values shown in the analysis) and calculate the difference between them, corresponding to the estimate of the intention to treat. Intention to treat (ITT) estimates the difference in outcomes between units selected for the treatment group and units selected for the comparison group, regardless of whether the units allocated to the treatment group receive the treatment [9]. The second step was to find the estimate of the LATE (local average treatment effect) by dividing the ITT by the Δ% of participation between the groups. The LATE determines an estimate of the local effect on a specific population subgroup; the type of group that participates is encouraged [9]. The theoretical model representation is shown in Table 3 below.

## 4. Results

Table 4 shows that group 1, which was encouraged to participate in the telemedicine program, had a participation rate of 87.5%. In contrast, group 2, which was not encouraged to participate in the program, had a participation rate of only 50%. We calculated the differences in the hospital admission authorization measures from 2020 to 2021 for the two groups. We can see that the differences were negative, −0.00045 and −0.06911, respectively, for AIH and outpatient care. This suggests that the group encouraged to adopt telemedicine had a lower hospital admission authorization rate. This is an essential result because during the pandemic, hospitals were full, and the health system collapsed without being able to cope with all the people who came to the health system. More specific triage through telemedicine allowed people with more significant needs to be sent to hospitals, and people who did not urgently need treatment were not encouraged to seek out these already overloaded hospitals.

The impact of telemedicine on outpatient care is equally noteworthy. The results show a significant difference of −0.0691 from telemedicine in 2020, which marked a reduction in the use of outpatient clinics during the pandemic. This reduction in outpatient clinic usage, which was also overwhelming during this period, allowed for better care for those most in need. By encouraging the use of telemedicine, we were able to significantly alleviate the strain on the population, with a reduction in hospitalizations and congestion in hospitals. It is important to highlight that Brazil experienced a high number of COVID-19 deaths during this period, and the health system was unable to meet the demand for hospital care. 

In Table 4, for each group, the monthly number of procedures per federation unity (UF) in the period was calculated (sum of procedures per UF per year/total months of the period), followed by the overall monthly average for the group (sum of monthly values per state/8). Afterward, the value of procedures per inhabitant was calculated for each UF (total procedures per UF per year/projection of inhabitants per state in the corresponding year). Then, the average was calculated for each UF in the period (sum of procedures per inhabitant/number of occurrences). The group means were calculated by the sum of means per UF divided by eight. The projection of inhabitants for each state was extracted from IBGE data on the Brazilian population. The results are presented in Table 4 below.

The difference found after the program’s implementation needs to be adjusted to see the program’s impact on the motivated group. Considering that the selection of indicators was restricted to clinical procedures (primary and hospital care) affected by teleconsultation and considering that the reduction in values may have been due to the adherence of states to the program, the impact of promoting hospitalizations (AIH) was −0.00119. The mean local effect estimate for the motivated group (LATE) is the result of dividing the intention to treat (ITT) estimate by the motivated group adherence difference [9], which was −0.00045/0.375 = −0.0012. The same occurred for outpatient care, remaining at –0.1843 <−0.06911/0.375>.

If we take the state of Pernambuco as an example, with a total of approximately 9700 thousand inhabitants, this means a savings of 11,640 (9,700,000 × −0.0012 = −11,543) hospital procedures and 1788 (9,700,000 × −0.1843 = −1788) outpatient visits. This state accounted for 2979 clinical procedures through teleconsultation. Across the northeast region, there were 14,247 teleconsultations. In the north region, there were only 728. In Brazil, there were 292,251 teleconsultations in the SUS until April 2021.

As for the cost of the procedures (according to Table 5 below), both teleconsultation and face-to-face consultations have the same value, according to data from DataSus/Sigtap. To verify the economic criterion, it would be necessary to determine the operational costs of the two modalities, which is different from the aims of this work. Therefore, it is not possible to compare the cost savings indicator through this criterion alone. Another indicator is that the evolution of COVID-19 occurred throughout the country with oscillations between states, but equally affected all regions, not being a differential factor for participation in the program.

## 5. Discussion

The methodology used to evaluate the telemedicine/teleconsultation policy was applied to specific groups, and it was not possible to expand the results to the entire country. However, considering that the north and northeast regions have the highest rates of disability, the most significant demand is for applying public policies to improve and expand health services. It is estimated that participation was low in the north region and part of the northeast region, possibly due to the lack of encouragement and dissemination of accessible resources. According to the CETIC.br report [40], the most available services in public health units were remote monitoring and teleconsultation. Among the analyzed groups, indicator values were reduced concerning the baseline and greater use of telemedicine tools in group 1. It was also verified that the per capita use of SUS resources was more significant in group 2, demonstrating that they depended more on the public health system.

The work carried out by university hospitals during the pandemic deserves to be highlighted. The documentary research surveys demonstrated that the engagement of federal universities helped to encourage the use of the tools. According to the Ministry of Health website, news dated 26 May 2021 [41], teleassistance has become an alternative in the period of the COVID-19 crisis in university hospitals (HUs) administered by Empresa Brasileira de Serviços Hospitalares (Ebserh), a public company linked to the Ministry of Education (MEC) created by Law No. 12,550/2011 to manage university hospitals. Only the hospitals linked to the Federal University of Rio de Janeiro (a total of eight hospitals), the Clinical Hospital of the Federal University of Rio Grande do Sul, and the University Hospital of the Federal University of São Paulo are not administered by Ebserh [42].

The Clinical Hospital in Recife, linked to the Federal University of Pernambuco (UFPE), developed a platform for teleguidance via chat and schedules teleconsultations via videoconference [42,43]. Through its UFPE Telehealth Center (Nutes), HC/UFPE performed 12,869 virtual consultations during the COVID-19 pandemic, of which 9543 were between teleconsultations and tele-orientations, and the remaining 3326 were telediagnoses.

The Ebserh/MEC Network hospital complex in Fortaleza (CH-UFC/Ebserh/MEC) is another example of using technology for virtual patient care. According to Ebserh [43], the center has a telehealth unit comprising four virtual offices equipped with computers, headphones, headsets, and webcams to meet the need for consultations and remote care.

The Lauro Wanderley University Hospital (HULW) of the Federal University of Paraíba created a call center that received more than a thousand calls in 15 days of operation, according to Ebserh, which manages the unit [44]. The hospital, like others, suspended face-to-face consultations due to the pandemic and began operating the remote service on 18 May 2020. Consultations are carried out via WhatsApp or the Teams platform. The result was so positive that the HULW considered maintaining the system after the pandemic [44]. The same happened with the University Hospital of the Federal University of Sergipe (HU-UFS). According to the UFS [45], the disclosure of the system and telephone numbers for assistance was made by employees through direct contact with those people who were already patients at the University Hospital and through the media. The Federal University of Bahia, in partnership with Fiocruz, created the Tele Coronavirus service accessed by number 155 with the support of the State Government. According to the Health Department of Bahia, the four state universities (Uneb, Uesc, Uefs, and Uesb), the Bahiana School of Medicine, FTC Salvador, Unifacs, Federal University of Recôncavo da Bahia, Federal University of Southern Bahia, and Fesftech, the developer of the platform, joined the initiative. Volunteers from medical schools carried out the work [46].

There are now 50 university hospitals linked to 35 federal universities, 40 administered by Ebserh. Research on the efficiency of care in the 40 HUs showed that among the ten with maximum efficiency, six are in the northeast region, one is in the north region, and the others are in the south and southeast regions [47].

On 14 April 2020, MS reported that 5.7 million people had already sought TeleSUS services, and of this total, 2.4 million had symptoms of the coronavirus [48]. The goal was to contact 120 million Brazilians through Busca Ativa, and since 1 April, when TeleSUS was launched, 2.2 million automatic connections had already been made. The numbers published on 23 June 2020, by MS, totaled 73.3 million people throughout Brazil served by TeleSUS since April: 25 million completed the service; 71% showed improvement; 22% remained stable; 7% had worsened health status; 1.8 million people had teleconsultations with health professionals [49].

Telemedicine was also widely used in the private sector, demonstrating that the main benefits of telemedicine pointed out by respondents by Capterra in 2020 were the possibility of accessing treatment from anywhere (72%) and the lower risk of contamination (71%) [50].

A February 2020 survey of 2258 Brazilian doctors from 55 specialties showed that 89.81% of respondents believe in the benefits for the SUS of using modern digital tools, and 90.21% of doctors consider that new digital technologies, with a high standard of safety and ethics, can help improve the health of the population [4].

The Internet Steering Committee in Brazil disclosed that 94% of basic health units (UBSs) had a computer, 89% used electronic health systems (78% in 2019), and 53% used profiles on social networks (46% in 2019) [40]. ANATEL corroborated these aspects since the improvement in telecommunications networks, remote work tools, and distance learning were among the primary responses of the telecommunications sector to the COVID-19 pandemic [51].

Our methodology consisted of evaluating whether the policy of promoting the use of telemedicine has been effective in Brazil during the pandemic. By creating a policy to encourage telemedicine, we determined that some people continue to go to hospitals and do not adhere to telemedicine. In contrast, others have stopped going to hospitals and always adhere to the policy. As a result, the policy had no impact on two groups of people. We estimated how many people will only participate and adhere to telemedicine treatments if they are encouraged to do so. We showed that the demand for telemedicine care was high in the Brazilian states where there was this stimulus, even after controlling for both types of possible errors (endogeneity control). According to Gertler [9], “random promotion is a creative strategy that generates the equivalent of a comparison group for impact assessment purposes” (p. 115). Nevertheless, it allows for the impacts of public policies to be assessed assertively, which can be essential in the public policy cycle to discuss and re-evaluate their impacts.

## 6. Conclusions

This study reached the following conclusions: 1—It is not possible to expand the evaluation results to the entire SUS population, but we identified that the north and northeast regions strongly demand public policies to improve and expand health services; 2—The participation of the north region and the MA and PI states of the northeast region was low, possibly due to the lack of encouragement and dissemination of the available resources, remembering that the responsibility of the health system is shared by the three federative spheres (noting that TeleSUS can be accessed from a cell phone and the call is free); 3—Federal universities helped to encourage the use of tools; 4—A reduction in the values of the indicators was observed concerning the baseline and more meaningful use of telemedicine tools by the states of group 1; 5—The per capita use of SUS resources has been more significant in the states of group 2, demonstrating that these are more dependent on the SUS.

The expectation, in general, is that telemedicine is part of the solution and can be seen as a measure to reduce inequality in medical care in the country. The potential is immense, but efficient regulation is essential to curb fraud that could harm the population. Qualitative research already carried out, as mentioned in this article, has shown that healthcare professionals, policymakers, and telemedicine users await adequate regulation to ensure the safety of patients and physicians. See Silva et al. [52] for a discussion on forecasting the pandemic and Tabak et al. [53] for discussing health literacy and public health literature. Further research could explore how telemedicine helped reduce or limit contagion during the pandemic and the effects health literacy has on the effectiveness of telemedicine.

Although the implementation of the telemedicine policy was rushed in Brazil to avoid further contamination of people by COVID-19, we showed in this paper that it was an effective public policy. Many people have embraced telemedicine, reducing the pressure to seek care in hospitals when the health system was on the verge of collapse due to high demand and the inability to care for all those seeking care in hospitals. Through a public policy to encourage telemedicine, it was possible to reduce this pressure on the health system and better manage one of the biggest health crises ever to hit the country. Our results suggest that it is possible to stimulate telemedicine with relative success in times of crisis, a lesson that may be important for other countries.

The limitation of this research is that it uses secondary data, which depends on updating the information bases. As a recommendation for future similar research works, we propose to expand the indicators, generating a more extensive scope of verification. Another suggestion is evaluating the costs of telemedicine/teleconsultation in SUS services, including an analysis of operational and process costs with an impact on reducing service queues and savings for public coffers. An analysis of costs and benefits in line with the economic analysis of law can help to improve this service model [54].

Our results suggest that more research is needed to evaluate the impact of public health promotion policies. Reducing the risks associated with policies to encourage the use of telemedicine is essential. Further research could focus on different dimensions of the problem. One relevant question concerns the cost savings of adopting telemedicine programs. This aspect is crucial, especially in nations with limited resources where the state provides public health as a public good. Evaluating the relative costs and how they can help promote a more sustainable public health are relevant research objectives in the promotion of public health.

There are several concerns about the implementation of telemedicine due to a series of issues, such as a deterioration in the quality of medical care, possible fraud in care, and difficulty in accessing the internet in remote locations that may end up without medical assistance. The COVID-19 pandemic has required telemedicine to be implemented quickly so that medical care is not paralyzed. The public policy of implementing telemedicine in Brazil, a country of continental dimensions, has brought a series of important lessons that can be useful for other countries. Encouraging the use of telemedicine has worked well within Brazilian policy during the pandemic. Encouraging telemedicine can be used by other countries that need to increase healthcare coverage in their territories. This is particularly important in countries with difficulties providing healthcare throughout their territory, such as countries with spatial heterogeneity in healthcare.

Our article clarifies how Brazil’s telemedicine policy was implemented during the pandemic and its impacts. These results are relevant for public health promotion policies in other countries, which can use a similar design or the most relevant features appropriate to their economic, health, and cultural context. Other articles evaluating the impact of telemedicine promotion policies can help develop better and more effective public health promotion policies, taking into account differences between countries with regard to the structure of public health, cultural aspects, and other characteristics.

## Figures and Tables

**Table 1:** Institutional and Decision-making Structure of the SUS.

Decision Sphere	Deliberative Collegiate	Manager	Intermanagement Commissions	Representatives of Managers (*)
Federal	National Council (CNS)	Ministry of Health (MS)	Tripartite Commission (CIT)	CONASS CONASEMS
State	State Council (CES)	State Secretariats (SES)	Bipartite Commission (CIB)	
Municipal	City Council (CMS)	Municipal Secretariats (SMS)	Bipartite Commission (CIB)	COSEMS

Source: authors, based on information from the Ministry of Health. (*) National Council of the Secretary of Health (Conass): entity representing the state entities and the Federal District in the CIT; National Council of Municipal Health Secretariats (Conasems): entities representing municipal entities in the CIT; Council of Municipal Health Secretariats (Cosems): entities that represent municipal entities, at the state level, to deal with matters related to health, as long as they are institutionally linked to Conasems, in the way their statutes provide.

**Table 2:** Policy Indicators.

Indicators	Description	Measurement
Infrastructure	Availability of access links and equipment by the population	Number of hits; quality of the communication link; equipment X population
Process	The average population rate of use	Number of teleconsultations by region/state
Result (Clinical Care)	Primary care service Admissions Diagnostics	Number of primary and specialized consultations per person and by region/state: general, before and during the pandemic, and by teleconsultation
Economic	Cost of teleconsultation Cost–benefit impact	The total cost of teleconsultation versus the cost of face-to-face consultation
Complementary Data	Evolution of the coronavirus in Brazil population data	Number of cases per month; population growth forecast

Source: authors.

**Table 3:** Theoretical model of group distribution.

	Encouraged Group	Group Not (or Little) Encouraged	Impact
	**% of Participation = 87.5%** **MD Y = 88**	**% of Participation = 50%** **MD Y = 32**	**Δ% Participation = 37.5%** **ΔY = 56** **LATE = 56/0.375 = 149.33**
Never	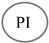	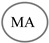	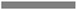
Participates if encouraged	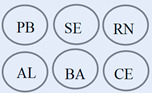	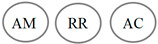	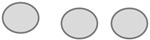
Always	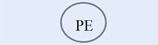	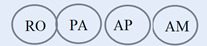	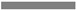

**Table 4:** Comparison of Means between Research Groups: Clinical Procedures.

	Group 1		Group 2			
	P/Month	By Inhabitant	P/Month	By Inhabitant	Difference	LATE
The General Average of AIH * Approved from 2020 to 2021	15,266	0.00236	8670	0.00280	−0.00045	−0.0012
**General Average Outpatient Care for 2020 and 2021**	1,824,183	0.28342	1,356,003	0.35253	−0.06911	−0.1843
**Participation Index**	87.5%		50%		37.5%	---

Source: authors, based on data collected from the DataSus database [www2.datasus.gov.br (accessed on 30 June 2021)]. * AIH—hospital admission authorization.

**Table 5:** Total Teleconsultations by Research Group between April/2020 and April/2021.

Teleconsultation				
2020–2021	301010307 *		301010315 **	
GROUP 1	1319	BRL 13,190.00	12,739	BRL 80,255.70
GROUP 2	271	BRL 2710.00	646	BRL 4069.80

Source: authors, based on data collected in the DataSus database (www2.datasus.gov.br, accessed on 30 June 2021). * Procedure: 03 01010307 medical teleconsultation in specialized care—BRL10.00 per consultation. ** Procedure: 03 01010315 teleconsultation by professionals with higher education in specialized care (except physician)—BRL6.30 per consultation. Note: also researched—Procedure: 0301010250 teleconsultations in primary care—BRL0.00.

## Data Availability

The data are contained in the article.
